# Spatial properties of astrocyte gap junction coupling in the rat hippocampus

**DOI:** 10.1098/rstb.2013.0600

**Published:** 2014-10-19

**Authors:** Stefanie Anders, Daniel Minge, Stephanie Griemsmann, Michel K. Herde, Christian Steinhäuser, Christian Henneberger

**Affiliations:** 1 Institute of Cellular Neurosciences, Medical School, University of Bonn, Bonn, Germany; 2 UCL Institute of Neurology, UCL, London, UK

**Keywords:** astrocytes, gap junction coupling, spatial properties, temperature, hippocampus, dentate gyrus

## Abstract

Gap junction coupling enables astrocytes to form large networks. Its strength determines how easily a signalling molecule diffuses through the network and how far a locally initiated signal can spread. Changes of coupling strength are well-documented during development and in response to various stimuli. Precise quantification of coupling is needed for studying such modifications and their functional consequences. We therefore explored spatial properties of astrocyte coupling in a model simulating dye loading of single astrocytes. Dye spread into the astrocyte network could be characterized by a coupling length constant and coupling anisotropy. In experiments, the fluorescent marker Alexa Fluor 594 was used to measure these parameters in CA1 and dentate gyrus of the rat hippocampus. Coupling did not differ between regions but showed a temperature-dependence, partially owing to changes of intracellular diffusivity, detected by measuring coupling length constants but not the more variable cell counts of dye-coupled astrocytes. We further found that coupling is anisotropic depending on distance to the pyramidal cell layer, which correlated with regional differences of astrocyte morphology. This demonstrates that applying these new analytical approaches provides useful quantitative information on gap junction coupling and its heterogeneity.

## 1. Introduction

A distinct feature of astrocytes is their extensive gap junction coupling [1–3]. It is thought to be essential for neuronal circuit function in many brain regions including the hippocampus. Gap-junction-coupled astrocyte networks in the hippocampal CA1 stratum radiatum rely on expression of connexin (Cx) 43 and 30 [3,4] and support neuronal function, for instance, metabolically [5]. Astrocyte network disruption in Cx43/30 double knockout mice modifies synaptic transmission and its plasticity at CA3–CA1 synapses [6] and impairs hippocampal potassium homoeostasis [4,6]. Decreased astrocyte coupling is also associated with a lower threshold for epileptiform activity *in vitro* [4], demonstrating its potential pathophysiological significance.

Gap junction coupling between astrocytes is heterogeneous throughout the brain [7,8]. In the rodent hippocampus, coupling can be detected immediately after birth and reaches adult levels by the middle of the second postnatal week [9,10]. Expression of Cx43/30 is not only developmentally regulated [11,12], but also affected in a number of diseases [13]. In addition to Cx43/30 expression, dynamic changes of astrocyte coupling [3] could also occur after connexin phosphorylation [14] and, indeed, activation of protein kinase C by phorbol esters was shown to reduce astrocyte coupling [9]. Astrocyte gap junction coupling is therefore not static, but dynamically regulated on several levels [3]. Understanding this regulation and its consequences for brain function requires experimental approaches that precisely quantify properties of gap junction coupling. In addition, assessment of gap junction coupling helps gauge how much of the astrocyte network is affected by experimental manipulations of single astrocytes [15].

A number of methods have been developed to study astrocyte gap junction coupling quantitatively. Electrophysiological evidence for coupling between astrocytes has been obtained by simultaneous patch clamp recordings from two astrocytes where a depolarizing current injected into one astrocyte also depolarizes the other [16,17]. Although double whole-cell patch clamp electrophysiology has superior resolution and is highly suitable for tests with acute manipulations it is also technically challenging and spatial aspects of coupling have to be extracted from multiple recordings. Optical methods often rely on diffusion of a fluorescent marker through gap junctions. For instance, a fluorescent and gap-junction-permeable dye can be bleached inside a single astrocyte. The recovery of fluorescence after photobleaching (FRAP) reflects the diffusion of dye back into the cell and is therefore a measure of gap junction coupling [8]. An inverse experimental approach is to load single astrocytes with a gap-junction-permeable dye, or other inert tracer, and visualize its spread into the astrocyte network after a defined time. This represents probably the most widely used approach [1,4,9,10,18,19]. Astrocyte networks of substantial size have been visualized by taking advantage of fast-diffusing tracers and/or ionotophoretic loading and allowing enough time for diffusion [4,9,20]. In these networks, hundreds of cells are gap-junction-coupled to a single astrocyte in CA1 stratum radiatum [4,9,20]. Often the visualized cells are then counted manually to estimate astrocyte coupling strength. This approach has been instrumental in establishing important fundamental properties of astrocyte gap junction coupling. It requires, however, minimization of any subjective bias during analysis and standardization of visualization and detection throughout a study. We therefore explored additional measures of dye coupling and its spatial properties. Dye spread in astrocyte networks was first simulated to identify suitable parameters. These were then tested on dye coupling data obtained from the hippocampal CA1 stratum radiatum and the molecular layer of the dentate gyrus, where fluorescent dye was loaded into astrocytes via whole-cell patch clamp pipettes and later visualized by two-photon excitation fluorescence microscopy.

## 2. Material and methods

Two-photon excitation fluorescence microscopy of astrocytes was performed as described previously [15,21] in slices obtained from Wistar rats and mice expressing EGFP under a GFAP promoter (hGFAP–EGFP mice). Briefly, 350 µm thick acute hippocampal slices were obtained from three to five week old rats and mice in full compliance with national and institutional guidelines on animal experimentation. Slices were prepared in an ice-cold slicing solution containing (in mM): NaCl 60, sucrose 105, KCl 2.5, MgCl_2_ 7, NaH_2_PO_4_ 1.25, ascorbic acid 1.3, sodium pyruvate 3, NaHCO_3_ 26, CaCl_2_ 0.5 and glucose 10 (osmolarity 300–305 mOsm l^−1^), and kept in the slicing solution at 34°C for 15 min before being stored at room temperature (RT, 21–23°C) in an extracellular solution containing (in mM) NaCl 126, KCl 2.5, MgSO_4_ 1.3, NaH_2_PO_4_ 1.25, NaHCO_3_ 26, CaCl_2_ 2 and glucose 10. This solution was also used for recordings at RT. Concentrations of NaHCO_3_ and NaCl were 21 and 131 mM for recordings at 34°C. pH of extracellular solutions was 7.35–7.45 at both temperatures (osmolarity adjusted to 295–305 mOsm l^−1^). Slices were allowed to rest for at least 60 min. For recordings, slices were transferred to a submersion-type recording chamber and superperfused with extracellular solution at RT or 34°C as indicated. All solutions were continuously bubbled with 95% O_2_/5% CO_2_.

Whole-cell recordings from astrocytes were obtained using standard patch pipettes (3–4 MΩ) filled with an intracellular solution containing (in mM) KCH_3_O_3_S 135, HEPES 10, di-Tris-phosphocreatine 10, MgCl_2_ 4, Na_2_-ATP 4, Na-GTP 0.4 (pH adjusted to 7.2 using KOH at RT, pH approximately 7.1 at 34°C, osmolarity 290–295 mOsm l^−1^). Although Cx43-containing gap junctions show little sensitivity to pH between 7.0 and 7.3 [22], it is possible that temperature-dependent changes of intracellular pH, potentially occurring in coupled cells distant to the patched cell, could have marginally affected dye coupling. Membrane-impermeable dyes Oregon Green 488 BAPTA-1 (200 µM, Invitrogen) and Alexa Fluor 594 hydrazide (20–40 μM, Invitrogen) were routinely added to the intracellular solution. Passive astrocytes were identified by their small soma size (approx. 10 μm), low resting potential (less than −80 mV without correction for the liquid-junction potential), low input resistance (less than 10 MΩ, current clamp), passive (ohmic) whole-cell current patterns, characteristic morphology and dye coupling (visualized in the Alexa emission channel). Astrocytes were either held in voltage clamp mode at their resting membrane potential or in current clamp.

Astrocytes and gap-junction-coupled networks were visualized by two-photon excitation fluorescence microscopy. We used a Scientifica two-photon system (Scientifica UK) and a FV10MP imaging system (Olympus) optically linked to a femtosecond pulse laser Vision S (Coherent, *λ* = 800 nm) both integrated with patch-clamp electrophysiology (Multiclamp 700B, Molecular Devices). Setups were equipped with 25× (NA 1.05) and 40× (NA 0.8) objectives (Olympus). The laser power was adjusted to 3–6 mW under the objective. Once in whole-cell mode, Alexa Fluor 594 typically equilibrated across the astrocyte arbour within 5 min. To ensure stable and rapid dye filling whole-cell access resistance (*R*_A_) was monitored throughout. Recordings with an initial *R*_A_ above 20 MΩ (on average 11.2 ± 0.9, *n* = 12) or *R*_A_ changes of more than 30% were discarded. After 20 min of dye loading a single image stack was obtained (512 × 512 pixels, less than 0.7 µm per pixel, stack size greater than 200 × 200 × 80 µm^3^).

Analysis of gap-junction-coupled networks was performed in ImageJ (NIH), Origin (OriginLab) and Matlab (Mathworks). Gap-junction-coupled cells were manually identified in *x–y–z* image stacks. Their fluorescence intensities were determined in regions of interest (5 × 5 µm^2^) centred on the soma and corrected for background fluorescence. Fluorescence intensity of a probe decayed monoexponentially with depth in the tissue (intensity *I*(*z*) = *I*_0_ exp(−*z*/*k*), *k* = 44.1 ± 2.1 µm, *n* = 3). Cell intensities were corrected for depth below the slice surface accordingly. Positions (in µm) of dye-coupled cells were calculated relative to the patched cell. Their intensities were normalized to the patched cell. Data of individual image stacks (*x_i_, y_i_, z_i_, I_i_*), with the patched cell at (0, 0, 0, *I*_0_), were stored for further analysis as described in §3. For analysis of coupling anisotropy (*C*_A_) and distance of centre of mass (*D*_CM_), the *z*-position was ignored and the dataset rotated in the *x–y* plane around the *z*-axis such that the *x*-axis was always parallel to the pyramidal cell layer (see §3*a*).

Simulations of astrocyte dye coupling were performed in Matlab. Astrocytes were modelled as single compartments and arranged in a three-dimensional grid (20 × 20 × 20 cells) separated by 40 µm in each dimension (equivalent to a numerical density of approx. 15 000 mm^−3^, estimated from previously performed astrocyte labellings [15]). The fluorescent dye concentration was represented as a percentage of the intra-pipette concentration. Whole-cell patch clamp and dialysis of a single astrocyte in the network were simulated by keeping the dye concentration (*C*) in the centre cell at 100% throughout the simulation. Dye coupling between cells was described by a coupling rate (*C*_R_ in s^−1^). *C*_R_ was fixed between each cell and its six immediate neighbours at the beginning of a simulation and was symmetric between cells. At each time point (20 min, time step 2.5 s), dye flux between all connected cells was calculated by −Δ*C C*_R_ and concentrations adjusted accordingly. *C*_R_ was adjusted to obtain dye coupling similar to experimental data.

FRAP of EGFP was used to gauge intracellular diffusivity [23]. FRAP was measured by line scanning across astrocyte cross sections (figure 2*f*) at 1–3 ms per line. Bleaching was induced by increasing the laser power at the objective to 15–30 mW for 500 ms. EGFP fluorescence was allowed to recover by closing the laser shutter for 1 s (figure 2*g*). FRAP was analysed as illustrated (figure 2*g*).

Morphology of EGFP-expressing astrocytes was analysed in Matlab (figure 3*c–e*). Fluorescence images of individual astrocytes from CA1 stratum radiatum were rotated so that their *x*-axis was in parallel with the pyramidal cell layer and background-corrected. Fluorescence intensity was normalized to somatic values. Astrocyte territories were then divided into four sectors by placing two diagonal lines intersecting at the centre of the soma (see schematic in figure 3*c*, dotted lines). Orientation-specific analysis was performed by calculating the area and average fluorescence intensity of the sectors parallel to the pyramidal cell layer (figure 3*c*, red horizontal sectors, *A_X_* and *F_X_*) and perpendicular (figure 3*c*, blue vertical sectors, *A_Y_* and *F_Y_*). Circular astrocyte territories would have a ratio *A_Y_/A_X_* of unity, whereas *A_Y_/A_X_* > 1 would indicate territories elongated perpendicular to the pyramidal cell layer (figure 3*c,d*). Astrocytes were categorized as ‘close to the pyramidal cell layer’ when their soma was not further away from the pyramidal cell layer than half of the thickness of the stratum radiatum and as ‘distal’ otherwise.

Numerical data are reported as mean ± s.e.m. or ±CI (figure 2*d*) with *n* being the number of samples. In figures, asterisks indicate statistical significance (details in figure or legend). Student's *t*-tests and others were used as indicated.

## 3. Results

### (a) Quantification of dye coupling in simulated astrocyte networks

**Figure 1.**
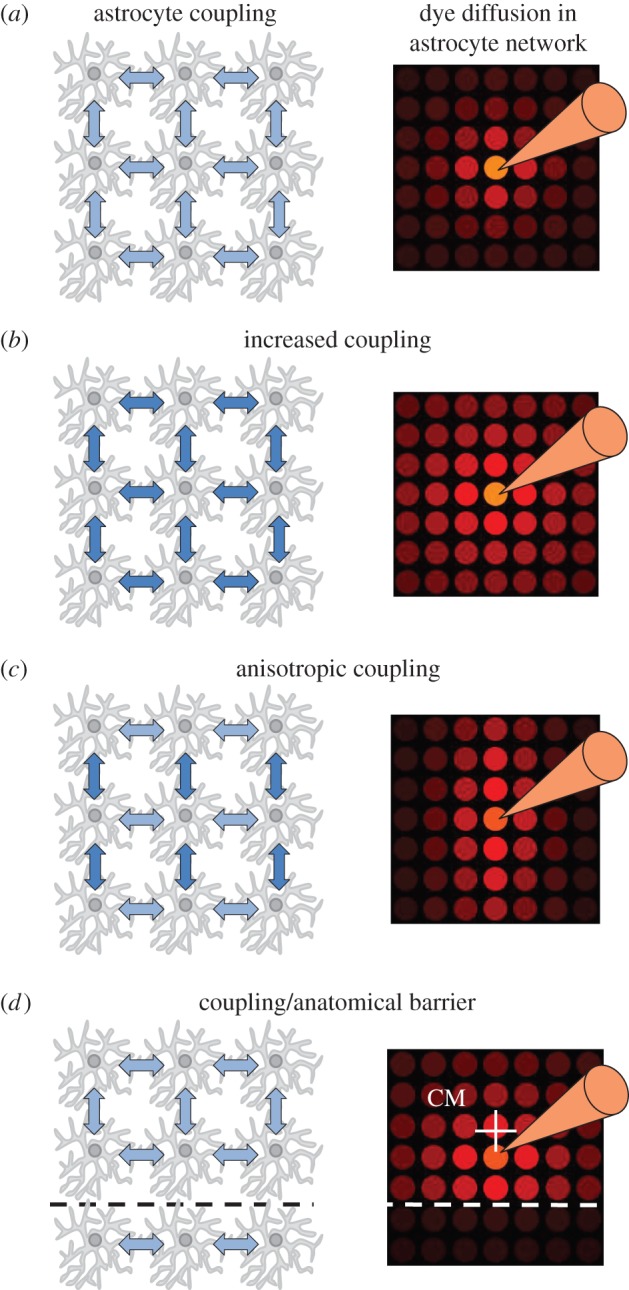
Changes of astrocyte coupling affect the spread of dye injected into a single astrocyte. Diffusion of dye from a simulated patched astrocyte (pipette) in a three-dimensional network was simulated in several conditions (left column, coupling represented by arrows, coupling strength by colour intensity). A single plane of the simulated network containing the patched cell is displayed (right column). Each circle represents a single cell. Dye concentration is colour-coded with bright colours corresponding to high concentrations. (*a*) Homogeneous coupling between astrocytes results in symmetric dye concentrations around the patched cell. Dye concentrations in gap-junction-coupled astrocytes decrease with increasing distance. (*b*) Enhancement of coupling between astrocytes increases the spread of injected dye into the network. (*c*) Increasing coupling along a specific axis, i.e. anisotropic coupling, results in enhanced dye spread in the astrocyte network along this axis (*y*-axis in this example). (*d*) Introducing a barrier with limited coupling (e.g. at an anatomical border, dashed line) increases dye spread along and away from that barrier. The shift of the centre of mass (CM, white cross) away from the patched astrocyte reflects the asymmetry of the coupled astrocyte network under these conditions (compared with panel *a*). (Online version in colour.)

We first explored dye diffusion from a single patched astrocyte into the gap-junction-coupled network in a model (§2 Material and methods). When astrocyte dye coupling rates were homogeneous throughout the network, dye spread symmetrically into the tissue (figure 1*a*) and the cellular dye concentration decayed monoexponentially with distance (*d*) from the patched cell (*C*(*d*) = *C*_0_ exp(−*d*/*C_*λ*_*), coupling length constant *C_*λ*_* = 30 µm, *R*^2^ = 0.99). Strengthening of dye coupling by scaling coupling rates by a factor of 4 increased dye diffusion into the modelled astrocyte network (figure 1*b*) and increased the coupling length constant *C_*λ*_* to 39 µm (*R*^2^ = 0.92). This indicates that the coupling length constant covaries with the coupling strength between cells but is not proportional to it. Nonetheless, obtaining the coupling length constant experimentally could provide a measure of overall strength of coupling in astrocyte networks *in situ*.

Coupling in astrocyte networks may not, however, be homogeneous. Astrocytes in the CA1 stratum radiatum, for example, are oriented such that their larger branches are in parallel to pyramidal cell apical dendrites [24]. This was speculated to promote diffusion in the astrocyte network along the same axis and to support potassium homoeostasis [4]. We therefore tested how directional preferences of coupling affect dye spread in the astrocyte network in the model by lowering the coupling strength along the *x*-axis by 80% (figure 1*c*). As expected, this favoured dye spread into the network in other directions giving the cloud of strongly visible cells an ellipsoid shape (figure 1*c*). This anisotropy of dye spread (*C*_A_, coupling anisotropy) can be quantified in two dimensions by *C*_A_ = Σ|*C_i_y_i_*|/Σ|*C_i_x_i_*|, where *C_i_, x_i_* and *y_i_* are the dye concentrations and positions relative to the patched cell. In the simulated example, *C*_A_ was 2.34 (figure 1*c*), whereas it was 1.0 for symmetric coupling illustrated in figure 1*a,b*. Experimental analysis of the coupling anisotropy could therefore reveal directional preferences of diffusion in astrocyte networks.

Preferential coupling in one direction could also result from disruption or strong reduction of gap junction coupling at anatomical borders. Such a scenario was implemented in the model by reducing coupling by 90% along an *x–z* plane close to the patched cell (figure 1*d*, dashed line) while keeping coupling homogeneous everywhere else. Consequently, dye loaded into the patched cell tended to diffuse asymmetrically away from the anatomical border and the patched astrocyte (figure 1*d*). The visual impression is a displacement of cells containing higher dye concentrations away from the patched cell. This can be quantified by calculating the centre of mass (white cross in figure 1*d*). Its distance *D*_CM_ to the patched cell is given by 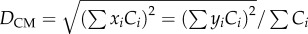 and is 33.9 µm for the example shown in figure 1*d*. At the same time, the coupling anisotropy was 0.82 indicating that dye also tends to diffuse along the introduced border (figure 1*d*, dashed line).

### (b) Strength of dye coupling

**Figure 2.**
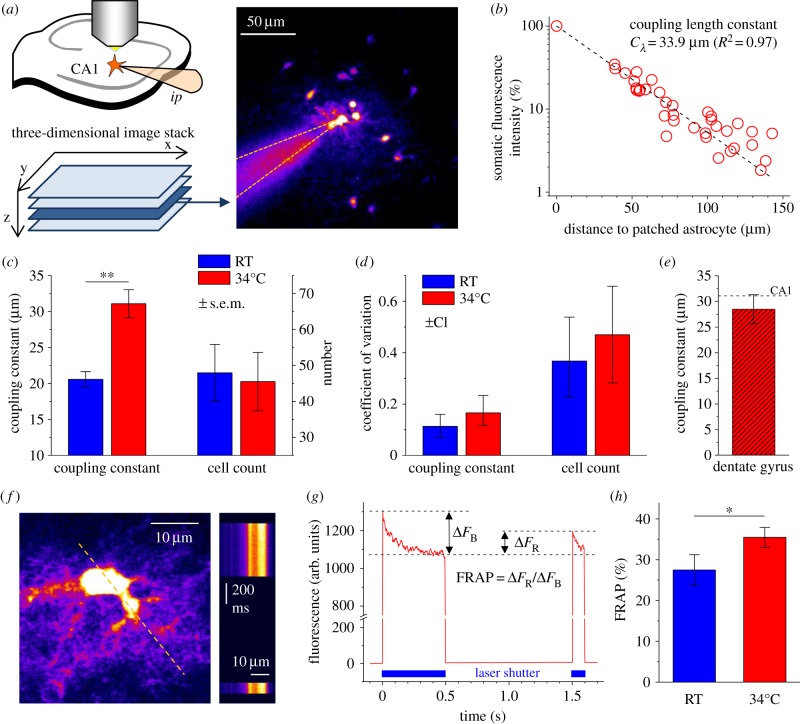
Visualization and quantification of astrocyte coupling in CA1 stratum radiatum. (*a*) Astrocytes were held in the whole-cell patch clamp configuration and dialysed with the fluorescent dye Alexa Fluor 594 via the patch pipette (*ip*, top left panel). *X–y–z* image stacks were obtained after 20 min and analysed. Right panel: sample slice of an image stack containing the patched astrocyte (dashed lines, patch pipette). (*b*) Somatic fluorescence intensity of gap-junction-coupled cells was normalized to the patched cell and corrected for depth within the slice (same recording as in (*a*), 36 cells). The normalized intensity (*I*) decreases monoexponentially with distance (*d*) from the patched cell (dashed line, monoexponential fit). The coupling length constant *C_*λ*_*, *I*(*d*) = *I*_0_ exp(−*d*/*C_*λ*_*) was used to quantify fluorescent dye spread into the gap-junction-coupled network. (*c*) Astroglial networks were studied at RT (*n* = 5) and at 34°C (*n* = 7). *C_*λ*_* was temperature-sensitive (unpaired *t*-test, *p* = 0.0011) whereas manually determined cell counts were not (*p* = 0.83, error bars are s.e.m.). (*d*) Coefficients of variation indicate a lower variability of C_λ_ analysis (error bars indicate 5–95% confidence intervals obtained by bootstrap analysis). (*e*) A *C_*λ*_* similar to CA1 stratum radiatum was observed in the molecular layer of the dentate gyrus (*n* = 18, *p* = 0.46, unpaired *t*-test, dashed line for comparison with CA1). FRAP of EGFP expressed by astrocytes was used to gauge intracellular diffusivity. Bleaching was induced by high power line scanning (*f*, left panel, dashed line). Fluorescence recovery occurred when unbleached EGFP diffused into the imaged region while the laser shutter was closed (1 s, *f*, right panel, *g*, blue bars indicate laser exposure). The bleached fraction of fluorescence Δ*F*_B_ and the recovered fraction Δ*F*_R_ were measured and used to quantify FRAP = Δ*F*_R_/Δ*F*_B_ (*g*). FRAP was significantly stronger at 34°C than at RT (*h*_*i*_, *n* = 10 and 12 for RT and 34°C respectively, unpaired *t*-test, *p* = 0.048). (Online version in colour.)

The practical usefulness of the coupling length constant, coupling anisotropy and centre of mass displacement was tested experimentally on astrocyte networks in rat CA1 stratum radiatum. Astrocytes were filled with the fluorescent indicator Alexa Fluor 594 via the patch pipette (figure 2*a*; §2 Material and methods). After 20 min of dye filling, a single image stack was obtained. The positions of gap-junction-coupled cells relative to the patched cell and their somatic fluorescence normalized to the patched cell were recorded. The relationship between the three-dimensional distance of coupled cells to the patched cell and their relative fluorescence intensity was approximated by a monoexponentially decaying function to obtain the coupling length constant (*C_*λ*_*; figure 2*b*). We took advantage of the temperature dependence of gap junction conductance [25] to test whether changes of coupling can be detected by measuring *C_*λ*_*. Astrocyte networks filled with dye at RT had a coupling length constant of 20.6 ± 1.1 µm (*n* = 5). In contrast, *C_*λ*_* was increased when experiments were performed at 34°C (31.1 ± 1.9 µm, *n* = 7, unpaired *t*-test *p* = 0.0011). For comparison, cells dye-coupled to the patched astrocyte were also manually counted in the same image stacks. Surprisingly, cell counts did not show a temperature dependence (RT, 48 ± 7.9 cells, *n* = 5, 34°C, 45.6 ± 8.1 cells, *n* = 7, *p* = 0.83) probably owing to their high variability. The coefficient of variation (CV) of cell counts was 0.37 (RT) and 0.47 (34°C) and, interestingly, the variability of cell counts determined from experiments where astrocyte networks were biocytin-filled at RT was very similar (CV 0.38, not shown). In contrast, coupling length constant CVs were 0.11 (RT) and 0.17 (34°C, see figure 2*d* for confidence intervals). *C_*λ*_* is therefore a relatively sensitive measure of astrocyte coupling.

To compare coupling length constants (*C_*λ*_*) between different brain regions astrocyte coupling was also studied in the molecular layer of the dentate gyrus at 34°C. *C_*λ*_* was similar to CA1 stratum radiatum (figure 2*e*), and no significant differences between dorsal and ventral recordings were detected (ventral 26.9 ± 4.3 µm, dorsal 30.1 ± 3.9, *n* = 9 both groups, *p* = 0.58, unpaired *t*-test) indicating that the spatial extent of coupling could be homogeneous and does not follow the previously reported dorsal–ventral gradient of astrocyte numerical density in the molecular layer of the dentate gyrus [26].

The temperature-dependent increase of *C_*λ*_* was approximately 51% from RT to 34°C (in CA1) and reproducing a similar increase of dye coupling in the model required a more than threefold increase of the coupling strength. Temperature effects on gap junction function and intracellular diffusivity may underlie stronger coupling at 34°C. The temperature dependence of intracellular diffusivity was estimated by FRAP of gap-junction-impermeable cytosolic EGFP expressed by astrocytes (figure 2*f,g*, hGFAP–EGFP mice). FRAP increased by 28.8% from RT to 34°C (figure 2*g,h*) which is equivalent to increasing the diffusion coefficient by approximately 40% in a simplified model (electronic supplementary material). It therefore seems unlikely that increased dye coupling in experiments at 34°C is solely a consequence of faster Alexa Fluor 594 diffusion in the cytosol.

### (c) Spatial properties of dye coupling

**Figure 3.**
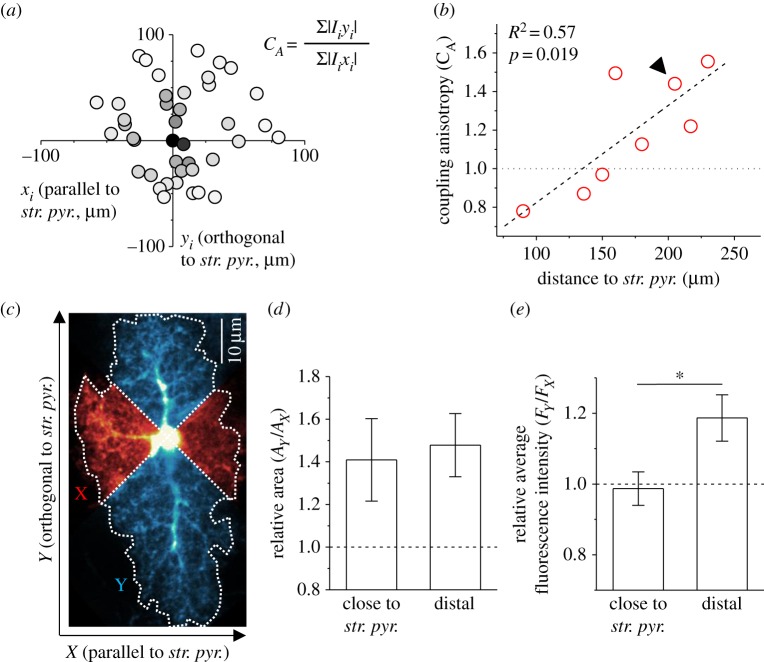
Astrocyte coupling is anisotropic in CA1 stratum radiatum. (*a*) Fluorescence intensities (*I_i_*) of gap-junction-coupled cells at positions (*x_i_, y_i_*) relative to the patched cell (single experiment, grey values represent *I_i_,* dark is high *I_i_*). The cloud of gap-junction-coupled cells was rotated around the *z*-axis such that the *x*-axis is parallel to the stratum pyramidale (*str. pyr.*). Note the localization of strongly coupled cells (dark) along the *y*-axis (orthogonal to the *str. pyr.*). Coupling anisotropy (*C*_A_) was calculated as defined (inset). (*b*) Coupling anisotropy depends on distance of the patched astrocyte from stratum pyramidale (*n* = 8, dashed line, linear fit). Arrowhead indicates the data point corresponding to the example shown in (*a*). Analysis of position-dependent astrocyte morphology was performed on EGFP-expressing astrocytes as illustrated (*c*, sample astrocyte). Optical cross sections of astrocytes were background-corrected, normalized to somatic fluorescence and subdivided into sectors parallel to the pyramidal cell layer (red, *X*) and perpendicular (blue, *Y*). Orientation preferences were quantified by calculating ratios of sector areas (*A_Y_/A_X_*) and their average fluorescence intensities (*F_Y_/F_X_*). Also see §2 Material and methods. (*d*) Area ratios showed a directional preference for the *y*-axis (*A_Y_/A_X_* > 1.0), i.e. perpendicular to *str. pyr,* close to the pyramidal cell layer (*n* = 19) and in more distal astrocytes (*n* = 17, *p* < 0.05, one-population *t*-tests, *p* = 0.78, two-population *t*-test). (*e*) Average fluorescence intensities ratios showed similar directional preferences only in astrocytes distal to the *str. pyr.* (*F_Y_/F_X_* > 1.0, same *n* as in *d*, *p* < 0.02, one and two-populations *t*-tests). (Online version in colour.)

Previous studies suggested that astrocyte morphology and orientation in CA1 stratum radiatum may favour diffusion in parallel to apical dendrites of CA1 pyramidal cells [4,24]. A fluorescent dye may therefore diffuse preferentially along this direction in the astrocyte network resulting in anisotropic dye coupling (figure 1*c*). At the same time, the CA1 pyramidal cell layer may represent an anatomical border at which gap junction coupling is reduced, and thus dye diffuses predominantly away and along the cell layer (figure 1*d*). We therefore analysed coupling anisotropy (*C*_A_) and distance of centre of mass (*D*_CM_) in a subset of image stacks in the *x–y* plane. (The *z*-positions of dye-coupled cells were ignored for calculation of *C*_A_ and *D*_CM_ in this context, because the pyramidal cell layer is perpendicular to the focal plane.) Because orientation of the pyramidal cell layer varies across experiments, data points (*x_i_, y_i_, I_i_*) were rotated around the *z*-axis such that the new *x*-axis is parallel to the pyramidal cell layer (figure 3*a*). The average *C*_A_ was 1.18 ± 0.10 (*n* = 8). Interestingly, we observed a positive correlation of *C*_A_ with the distance of the patched cell to the pyramidal cell layer (figure 3*b*, *p* = 0.019). Close to the pyramidal cell layer, the coupling anisotropy was below unity indicating dye spread in the astrocyte network preferentially in parallel to the pyramidal cell layer. In contrast, dye loaded into astrocytes more distant to the pyramidal cell layer tended to diffuse more perpendicular to the cell layer as indicated by coupling anisotropies bigger than unity. Such a correlation was not observed for *D*_CM_ (*p* = 0.71, not shown). *D*_CM_ for all eight image stacks combined was 3.2 µm which is about the soma radius of a rat astrocyte or less. This indicates a negligible displacement of the centre of mass of the dye-filled astrocyte population away from the patched cells.

We next tested whether local heterogeneity of astrocyte morphology may underlie the gradient of coupling anisotropy in CA1 stratum radiatum. Single EGFP-expressing astrocytes were imaged to visualize their morphology and analysed as illustrated (figure 3*c–e*, also see §2 Material and methods). We found that territories of astrocytes were elongated along an axis perpendicular to the pyramidal cell layer (figure 3*d*), irrespective of their distance to the pyramidal cell layer. The average fluorescence intensity of cytosolic EGFP was also higher along this axis but only in astrocytes distal to the pyramidal cell layer (figure 3*e*) where coupling was anisotropic (figure 3*b*). Because normalized fluorescence intensity of a cytosolic dye is a measure of astrocyte cytosol volume distribution [27], assuming homogeneous EGFP concentrations, this observation suggests that astrocyte processes are more abundant or thicker along an axis perpendicular to the pyramidal cell layer in these distal astrocytes. Such morphology could indeed promote diffusion along this axis and result in anisotropic astrocyte coupling.

## 4. Discussion

A model of dye diffusion from a single astrocyte into the gap-junction-coupled astrocyte network was used to identify parameters for quantifying spatial properties of astrocyte coupling. Experimental data of fluorescent dye diffusion from a patched astrocyte into the network were then obtained and analysed accordingly. The spatial extent of coupling was readily quantified by a coupling length constant in simulations and experiments. Coupling length constants were similar in CA1 stratum radiatum and the molecular layer of the dentate gyrus and along its dorsal–ventral axis. However, by varying the recording temperature, we could detect temperature-associated changes in astrocyte coupling strength. These were not observed when the strength of dye coupling was quantified by manual cell counting. Higher variability of manual cell counts compared with calculation of the coupling length constant is a probable reason. For instance, randomly omitting half of the dye-filled astrocytes will greatly affect cell counts but much less the coupling length constant obtained by monoexponential approximation. The coupling length constant could therefore help in identifying more subtle modifications, physiological or pathophysiological, of astrocyte coupling. Its temperature-dependent increase was approximately 51% from RT to 34°C. Increased cytosolic diffusivity and/or changes of gap junction conductance may underlie this behaviour. FRAP experiments and modelling revealed that increased diffusion may only partly account for stronger coupling at 34°C. It therefore appears plausible that other mechanisms, such as rapid modification of gap junction conductance or gating properties [25] by Cx43/30 phosphorylation, expression or trafficking, also contribute to the temperature dependency of gap junction coupling.

Coupling was found to be anisotropic in CA1 stratum radiatum. Astrocytes distal to the pyramidal cell layer preferentially coupled perpendicular to the cell layer (figure 3). A similar orientation of large astrocyte processes [24] could underlie this coupling pattern because high diameter branches may provide a low-tortuosity path for diffusing molecules. Indeed, we found that EGFP-expressing astrocytes occupy territories elongated along the same axis. More importantly, when average EGFP fluorescence intensity was used as a measure of cytosolic volume distribution [27], a similar orientation was detected. This implies that processes of these distal astrocytes were more abundant and/or thicker along an axis perpendicular to the pyramidal cell layer, which may in turn promote dye coupling along this axis and thereby coupling anisotropy. In contrast, dye loaded into astrocytes close to the pyramidal cell layer tended to diffuse more strongly in parallel to the pyramidal cell layer although the effect appeared less pronounced. This could suggest a moderate reduction in gap junction coupling across the pyramidal cell layer similar to juvenile mice [18] and/or reflect different distribution of cytosol volume compared with distal astrocytes. However, although these measures capture key features of astrocyte morphology a deeper understanding of the structural basis of coupling anisotropy will require detailed reconstruction of astrocytes and their gap junctions at high spatial resolution, e.g. using electron microscopy. Such experiments will also provide clues about the origin of the observed heterogeneity of astrocyte morphology (e.g. synapse densities, spine morphology). Together, our findings illustrate that diffusion of biologically relevant molecules may be anisotropic with varying directional preference even within a hippocampal subfield and possibly depending on differences of astrocyte morphology. It is clear, however, that spread of the inert and membrane-impermeable fluorescent indicator Alexa Fluor 594 used here is not influenced by the various cellular mechanisms that will be critical for signalling molecules such as cAMP and IP3 [3] or ions such as potassium. Additional aspects of gap junction coupling that cannot be resolved by monitoring dye diffusion through the astrocyte network include detection of coupling asymmetries between two cells by rectifying gap junctions or isolating the contribution of leak from the astrocyte network through hemichannels. Preliminary simulations regarding the latter indicate that introduction of dye leak could be indistinguishable from a reduction of cell to cell coupling. Furthermore, astrocytes establish gap junctions not only among themselves but also with oligodendrocytes [16]. While such panglial coupling appears to be less abundant in the hippocampus it is prominent in other brain regions such as the thalamus [19]. Thus, for a meaningful interpretation of results obtained from dye coupling experiments the degree of panglial coupling may need to be known.

We have combined modelling of astrocyte dye coupling and recording of fluorescent indicator diffusion in the astrocyte network to identify parameters of astrocyte coupling. The coupling length constant and coupling anisotropy were found to be sensitive spatial measures. We show that astrocyte coupling is temperature-dependent and anisotropic in the stratum radiatum of the hippocampal CA1 region. The described approach complements existing analytical tools and can be easily adapted to various brain regions and to *in vivo* preparations. It requires, however, a linear read-out of somatic dye concentrations. This needs to be ensured during data acquisition and may not be guaranteed when a tracer is visualized by secondary reactions (e.g. biocytin). If a structural reference, such as the CA1 pyramidal cell layer, is missing, coupling anisotropy could be determined in two or three dimensions by principal component analysis (PCA) using relative positions and intensities of coupled cells. For example, the ratio of components returned by PCA on modelled data is 1.0 for homogeneous and 19.1 for anisotropic coupling (figure 1*a*,*c*). These approaches should be useful for studying astrocyte coupling and its physiological significance quantitatively and for gauging how far-reaching manipulations of individual astrocytes are.

## Supplementary Material

Supplementary material
