# Supplementary material for: Spatial properties of astrocyte gap junction coupling in the rat hippocampus
Source: Philos Trans R Soc Lond B Biol Sci. 2014 Oct 19;369(1654):20130600. doi: 10.1098/rstb.2013.0600 (PMC4173286; doi:10.1098/rstb.2013.0600)
Supplement: Supplementary material [file rstb20130600supp1.pdf]

Supplementary material

Anders, S. *Et al.* 2014. Spatial properties of astrocyte gap junction coupling in the rat hippocampus.
Phil. Trans. R. Soc. B. **369**. doi: 10.1098/rstb.2013.0600

Modelling of fluorescence recovery after photobleaching (FRAP)

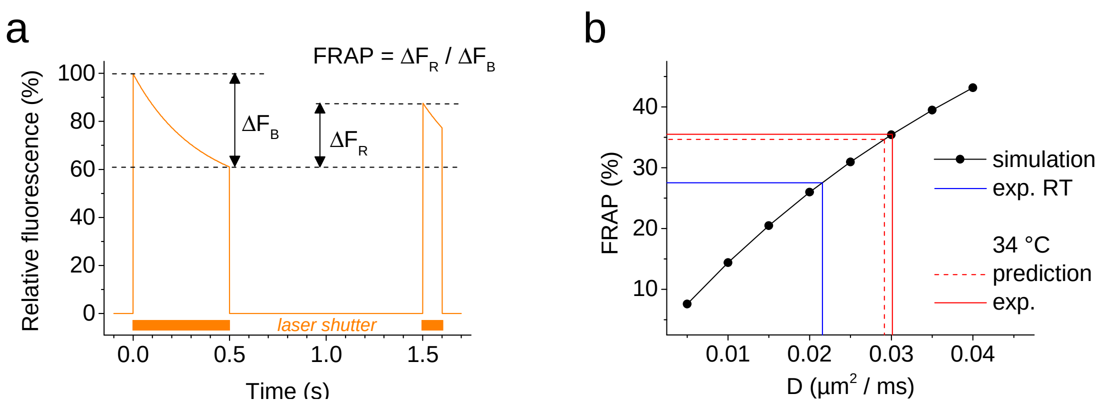

Figure S1: Modelling of fluorescence recovery after photobleaching (FRAP)

To gain a first insight into how experimental FRAP results correlate with changes of intracellular diffusivity we performed simulations of line-scanning FRAP in a continuous cubic volume (see below for details). A laser exposure protocol similar to experimental procedures was used (a, orange bars). FRAP was determined from simulated fluorescence traces as for experimental data (a). FRAP depends directly on the diffusion coefficient D in simulations (b, black dots). The experimentally observed FRAP of 27.5% at RT corresponds to D = 0.0216 µm^2/ms in simulations (blue lines), which is close to a reported EGFP diffusion coefficient of 0.025 µm^2/ms in HeLa cells using fluorescence correlation spectroscopy [1]. Increasing the temperature from RT to 34 °C is expected to increase D (according to the Stokes-Einstein equation, from 295 K to 307 K, assuming the viscosity of the intracellular medium to be equivalent to water [2,3]) by ~ 35% to 0.0292 µm^2/ms with corresponding FRAP of 34.6% in simulations (red dashed line). This is similar to the experimentally measured FRAP at 34 °C of 35.5% (red solid line). Therefore, the experimentally observed temperature-dependent increase of FRAP of EGFP in astrocytes (Figure 2g) can be estimated to reflect an increase of the EGFP diffusion coefficient by about 40%.

Details of FRAP modelling

A three-dimensional grid of cubic voxels (n, number of voxels along each dimension) was simulated. Dye flux between neighbouring voxels was calculated by Fick's first law. Photobleaching was implemented by reducing the dye concentration $c_{x,y,z}$ along a line at the centre of the simulated volume (diameter 1 voxel, length 50% of grid length, $x = \frac{n}{2}, y = \frac{n}{2}, z = \frac{1}{4}n \dots \frac{3}{4}n$) by $c_{x,y,z} \cdot r_{PB} \cdot \Delta t$ where r_{PB} is the photobleaching rate (in s^{-1}) and Δt is the time step

(in s). Voxel size was 0.5 x 0.5 x 0.5 µm^3 to approximate the point spread function of two-photon excitation imaging on the setup. An initial homogeneous dye concentration was set at the beginning of the simulation. The dye concentration was kept at its initial value at the borders of the simulated volume throughout.

Simulations ran for 1.8 seconds (-0.1 to 1.7 s). Laser exposure was simulated by enabling photobleaching from 0.0 to 0.5 s and 1.5 to 1.6 s (panel a, orange bars) allowing one second for recovery from photobleaching similar to experimental procedures (panel a, also see Figure 2). The fluorescence signal was considered equivalent to dye concentration at the bleached line during laser exposure and zero otherwise (panel a, orange trace). The bleached fluorescence ΔF_B and recovered fluorescence ΔF_R were analysed. FRAP was calculated as $\Delta F_R/\Delta F_B$. The time step Δt was reduced and n increased until further changes affected FRAP by less than 0.1 % (n = 15, Δt = 1 ms). FRAP was largely independent of changes of the photobleaching rate indicating that the kinetics of diffusion of unbleached dye into the imaged region is the primary determinant of FRAP magnitude. More than 10 fold changes of r_{PB} from 0.1 s^{-1} to 1.5 s^{-1} decreased FRAP by only 0.48 % (constant diffusion coefficient). r_{PB} was set to 0.435 s^{-1} to obtain similar bleached fractions (ΔF_B) as observed in imaging experiments (about 20 %).

1. Chen, Y., Müller, J. D., Ruan, Q. & Gratton, E. 2002 Molecular Brightness Characterization of EGFP In Vivo by Fluorescence Fluctuation Spectroscopy. *Biophys. J.* **82**, 133–144. (doi:10.1016/S0006-3495(02)75380-0)

2. Luby-Phelps, K., Mujumdar, S., Mujumdar, R. B., Ernst, L. A., Galbraith, W. & Waggoner, A. S. 1993 A novel fluorescence ratiometric method confirms the low solvent viscosity of the cytoplasm. *Biophys. J.* **65**, 236–242. (doi:10.1016/S0006-3495(93)81075-0)

3. Verkman, A. S. 2002 Solute and macromolecule diffusion in cellular aqueous compartments. *Trends Biochem. Sci.* **27**, 27–33.
